# Socioeconomic Differentials in Smoking Duration among Adult Male Smokers in China: Result from the 2006 China Health and Nutrition Survey

**DOI:** 10.1371/journal.pone.0117354

**Published:** 2015-01-09

**Authors:** Hong Guo, Zhihong Sa

**Affiliations:** 1 Independent Researcher, Beijing, China; 2 School of Social Development and Public Policy, Beijing Normal University, Beijing, China; Zhejiang University, CHINA

## Abstract

**Background:**

A smoker’s risk of diseases and death from smoking is closely related to his/her smoking duration. But little is known about the average length of smoking and the association between smoking duration and socio-economic status (SES) among Chinese smokers.

**Methods:**

A sample of male ever smokers (N = 2,637) aged 18+ years was drawn from the 2006 China Health and Nutrition Survey to examine the average length of smoking and socioeconomic differentials in smoking duration. Kaplan-Meier analysis was used to obtain median smoking duration. Log-logistic regression models were employed to estimate the relative duration of smoking, adjusted for demographic characteristics, smoking history, and health status.

**Results:**

Results showed that Chinese male ever smokers aged 18 years and older had a median duration of smoking of 58 years (95% *CI:* 56–61). Male ever smokers with a lower status job (i.e. farmers, manual and skilled workers, service workers, and office staff) had a significantly longer duration of smoking than those with a professional or administrative job after adjusted for demographic characteristics, smoking history, and health status. Individuals who earned the lowest income and who had no education or were being illiterate smoked for 11% and 14% longer, respectively, relative to those who had the highest income or who had college or above education.

**Conclusion:**

The findings demonstrated the problem of long smoking duration and a pattern of social disparities in smoking duration among Chinese male smokers. Social disparities in smoking behavior may exacerbate the already existing social inequalities in health. Thus, policies and interventions to promote smoking cessation should pay more attention to disadvantaged social groups.

## Introduction

With a population of 1.3 billion, China is the world’s largest producer and consumer of tobacco [1, 2], and bears a large proportion of deaths attributable to smoking worldwide. The 2010 China Global Adults Smoking Survey (GATS) reported that 53% of men aged 15 years and above are smokers; about the same number as in 2002 [3, 4]. The estimated number of deaths attributed to tobacco use has reached 0.8 million per year in China since 1990 and this number is expected to increase to 2 million by 2025 [5]. However, smoking cessation rates in China are very low-only 11% of smokers successfully quit, and 82% have never even thought about quitting [4].

Previous studies show that duration of smoking has a strong positive association with mortality and morbidities. Longer duration of smoking reduces life expectancy, and increases health risks such as lung cancer and coronary heart disease [6, 7, 8, 9]. For example, a study of US adults indicates that life expectancy of smokers who quit at 35 years exceeds that of continuing smokers by 6–9 years. Even among smokers who quit at 65 years, the life expectancy benefit is 1–3 years. Studies also show that the hazardous effects of lengthy smoking duration on death from lung cancer is even stronger than that of smoking intensity [10]. As smoking duration triples, lung cancer risk may increase as much as 100-fold [11]. Smoking duration is found to be an independent risk factor for chronic obstructive pulmonary disease (COPD) [12].

The vast amount of literature documents a consistent negative association between socioeconomic status (SES) and smoking in Western countries. A clear social gradient has been observed in smoking and smoking cessation by income, education attainment, occupation, and other measures of socioeconomic status [13, 14, 15]. A few studies examining the relationship between SES and smoking duration in Western countries indicate that individuals of lower SES have a longer duration of smoking, although findings varies with respect to specific measures of SES and in magnitudes of the association between SES and duration of smoking [16, 17, 18].

There are few studies on social differentiation in smoking in China, and most of these studies are descriptive. Similar to developed countries, existing studies on China show that individuals of lower educational attainment, income, and occupational status are more likely to smoke [19, 20, 21, 22], and less likely to quit smoking [23]. While lengthy years of smoking have detrimental health effects, to our knowledge, there is no study examining smoking duration and its association with SES among Chinese population.

Using 2006 China Health and Nutrition Survey (CHNS) data, the present study examined smoking duration and its association with SES among Chinese male smokers. The hypothesis was that male smokers of lower level of education, occupational status and income are more likely to have longer smoking duration after taking into account important confounding factors including age, smoking history, and health status.

## Methods

### Study sample

We used data from 2006 wave of the China Health and Nutrition Survey, an ongoing collaborative project of the Carolina Population Research Center at the University of North Carolina, the Institute of Nutrition and Food Safety at the Chinese Center for Disease Control and Prevention. Initiated in 1989, the longitudinal survey of CHNS was conducted in nine provinces and autonomous regions in China: Guangxi, Guizhou, Hunan, Hubei, Henan, Jiangsu, Shandong, Heilongjiang and Liaoning. Approximately one third of China’s population live in these provinces, which vary substantially in geography and socio-economic development. The sample has drawn on a stratified multi-stage cluster design whereby in each province, low-, middle-, and high-income counties were selected. The county seat and three randomly chosen villages were selected in each county subsequently. Also, in each province, the provincial capital, a low-income city, a suburban villages located near these cities were included. (A detailed description of the design of the CHNS can be found at http://www.cpc.unc.edu/projects/china). Although the CHNS data are not a representative sample of China’s population, previous studies show that the characteristics of the CHNS households and individuals are comparable to those from national samples [24, 25].

The CHNS collects extensive information on socio-economic status, health behaviors, and health outcomes which provide a good data source for the present study. In China, smoking prevalence is very high among males, and very low among females. There are also significant differences in determinants of men’s and women’s smoking behavior [26]. Our initial analysis showed similar gendered pattern in smoking prevalence (there were 2886 male ever smokers and 201 female ever smokers in the sample). The small sample size prevents reliable estimates of social correlates of smoking duration among women, whereas a pooled male and female sample may disguise the true relationships between smoking duration and socio-economic status. Therefore, we restricted our research sample and analysis to adult male smokers only.

Respondents who answered ‘‘yes’’ to ever smoking in their lifetime (N = 2,895) were initially included in the research sample. We then dropped cases with missing or uncertain values for smoking-related variables (i.e. current smoking status, age at smoking initiation, date of cessation; n = 119) and for covariates (n = 139) from the analysis. Our final analytic sample consisted of 2,637 male smokers aged 18 years and older.

### Measurement

The outcome variable was duration of smoking which was defined as number of years of daily smoking. Duration of smoking, i.e. time from smoking onset to cessation, was computed based on the questions ‘How old were you when you started to smoke (in years)?’ and ‘How long ago did you stop smoking (in months)?’. There might be some recalling errors in age of smoking onset. We therefore imputed age at smoking initiation as 10 for cases with a reporting of below ten years old (n = 12).

We used education attainment, current occupation and per capita family income as indicators of SES. Education was grouped into the following five categories based on respondents’ reporting of highest level of education and years of formal education: primary incomplete or no formal education, primary school, junior high school, senior high school or vocational school, and college or above. Occupation was divided into eight categories: farmer, skilled and manual worker, service worker, office staff, professional and administrator, other occupations, currently not employed, and being retired. Per capita family income from all sources was grouped into four categories by quartiles with the lowest income group as the first quartile, and the highest income as the fourth quartile. Other covariates of interest included smoking-related chronic diseases (yes = had any of the four types of chronic diseases including hypertension, diabetes, myocardial infarction and apoplexy), self rated health (good = excellent or good, not good = fair or poor), household registration status (rural and urban), age at initiation of smoking (≤18, 19–24, ≥25 years), respondents’ age (18–24, 25–34, 35–44, 45–54, 55–64, ≥65 years), marital status (married, unmarried including divorced, never married, and widowed), and region.

### Data analysis

The dependant variable of the study was duration of smoking from onset to cessation with current smokers at the time of the survey included as right-censored cases. We first presented description of sample characteristics and overall median duration of smoking. Then we presented results from bivariate analyses. Median duration of smoking was computed using Kaplan–Meier survival models. Bivariate associations between indicators of SES, covariates and duration of smoking were estimated using the log-rank test. Finally, univariate and multivariate log-logistic regression models were used to estimate the association between indicators of SES, other covariates and smoking duration before and after controlling for potential confounders. Cox proportional hazard regression models were initially employed to examine the association between SES and smoking duration. However, adjusted log–log survival curves and tests based on scaled Schoenfeld residuals suggested that the assumption of the Cox proportional model [27] was violated with regards to age, age of smoking initiation and occupation. We fitted three parametric models among the suitable distributions for accelerated failure-time (AFT) models: lognormal, log-logistic and Weibull regressions [28]. As the different distributional regressions have led to very similar estimates, we reported results obtained from the log-logistic regression models which is the most commonly used. Stata Version 9.0 was used to perform the analysis.

## Results

As shown in Table 1, a majority of male ever smokers in the sample were aged 45 years or older (59.3%), married (88.7%), or rural residents (59.3%). Most of them had a relatively low SES. While about three-fourth respondents had primary or less (36.4%)and junior high school (36.7%) education, only 26.9% had senior high school or above education. Only 12.0% had a white-collar job (professional/administrative jobs, or office staff), a majority of respondents were farmers (31.3%), manual and skilled workers (13.6%), or service workers (10.5%). 10.9% of the respondents reported having one or more chronic diseases, and more than one-third (37.2%) reported having fair or poor health status. While a majority of male ever smokers (80.3%) in the sample began to smoke regularly at 19 years or older, about one-fifth of them (19.6%) started to smoke at 18 years or younger (Table 1). Only 11.3% of male ever smokers reported having quitted smoking, and their median duration of smoking was 58 years (95%*CI*: 56–61).

**Table 1:** Sample Characteristics and Distribution of Smoking Duration among Male Ever Smokers Aged 18+, CHNS, 2006 (N = 2,637).

**Variables**	**% in sample (n)**	**% who have quit**	**Median duration of smoking in years^a^ (95% *CI*)**	**Log-rank test *x*^2^**
Overall	100.0 (2,637)	11.3	58 (56–61)	
Age (years)^b^				176.6 (*p*<0.001)
18–24	3.9 (102)	3.9	10 (10–11)	
25–34	12.6 (333)	5.1	22 (21–23)	
35–44	24.2 (638)	4.4	30 (29–30)	
45–54	24.9 (656)	7.2	40 (39–41)	
55–64	20.3 (536)	15.9	48 (47–49)	
>=65	14.1 (372)	31.2	57 (56–59)	
Marital status				0.0 (*p* = 0.96)
Married	88.7 (2,340)	11.2	58 (53–67)	
Unmarried	11.3 (297)	12.1	58 (56–62)	
Registration status				26.0 (*p*<0.001)
Urban	40.7 (1,074)	15.6	53 (50–57)	
Rural	59.3 (1,563)	8.3	68 (60-nc)	
Region				9.4 (*p* = 0.024)
Coastal	21.1 (556)	13.3	53 (51–59)	
Northeast	21.2 (559)	11.8	56 (48-nc)	
Inland	33.0 (870)	10.8	60 (58–63)	
Mountainous south	24.7 (652)	9.7	60 (54–68)	
Education				57.4 (*p*<0.001)
No education or illiterate	15.7 (413)	15.7	60 (58–62)	
Primary	20.7 (546）	10.2	56 (51-nc)	
Junior high	36.7 (969）	9.0	65 (50-nc)	
Senior high	19.4 (512）	11.4	55 (46-nc)	
College or above	7.5 (197)	15.8	46 (41-nc)	
Occupation^b^				47.6 (*p*<0.001)
Farmer	31.3 (830)	7.2	55 (53–57)	
Manual and skilled worker	13.6 (360)	4.7	45 (42–48)	
Service worker	10.5 (277)	3.6	49 (47–51)	
Office staff	3.3 (89)	5.7	44 (41–46)	
Professional and administrator	8.7 (234)	14.0	40 (38–43)	
Other occupations	4.5 (119)	9.3	47 (42–51)	
Unemployed	17.4 (471)	15.0	55 (53–58)	
Retired	10.7 (288)	33.3	53 (51–55)	
Income quartiles				31.4 *(p*<0.001)
Quartile 1	24.3 (640)	8.3	60 (58-nc)	
Quartile 2	24.9 (657)	9.1	58 (58-nc)	
Quartile 3	25.3 (667)	13.2	53 (48–57)	
Quartile 4	25.5 (673)	14.3	55 (48–67)	
Age of smoking initiation (years)				31.8 (*p*<0.001)
<=18	19.6 (708)	10.3	63 (60–71)	
19–24	53.6 (1,410)	10.9	58 (51–59)	
>=25	26.7 (519)	13.7	53 (47–55)	
Chronic disease				11.7 *(p*<0.001)
Yes	10.9(287)	28.9	52 (48–58)	
No	89.1(2350)	9.1	60 (58–62）	
Self rated health				0.08(*p* = 0.78)
Good/excellent	62.8(1657)	8.5	59 (56–65)	
Fair/poor	37.2(980)	16.0	58 (53–62)	

^a^ nc refers to not being computed due to ties and/or insufficient uncensored cases.

^b^ Due to ties and/or insufficient uncensored cases, we can’t compute median durations of smoking for occupation and age group. To compare results, we estimated mean durations of smoking for occupation and age group, which tend to be lower than median durations of smoking.

Results from the log-rank χ^2^ test showed significant differences in median years of smoking by education, occupation and income indicating individuals with lower SES smoked for longer durations (Table 1). Specifically, male ever smokers who were in each category of lower levels of education had a longer duration of smoking than those who had college or above education (55–65 vs. 46 years). Similarly, duration of smoking also showed a strong gradients with regards to occupations such that male ever smokers who were farmers (55 years), service workers (49 years), manual and skilled workers (45 years), office staff (44 years), all had a longer duration of smoking than those with professional or administrative jobs (40 years). Smokers who earned the lowest income smoked longer than those who earned the highest income (60 vs. 55 years). In addition, duration of smoking also varied by age of smoking initiation, chronic conditions, age, household registration status, and region at the *p* ≤ 0.05 level of significance.

Table 2 showed crude and adjusted time ratios associated with duration of smoking by various indicators of SES and covariates from log-logistic regression models. For ease of interpretation, we reported time ratios which are the exponentiated log-logistic regression coefficients. Time ratios can be interpreted as the factor by which smoking duration is different in one category compared with the reference category of a covariate [21, 12]. The crude results were very similar to the descriptive results reported in Table 1. The adjusted time ratios revealed some attenuation in the effect of socioeconomic status on smoking duration. After controlling for each other and all other covariates, education, occupation and income were still significantly associated with duration of smoking. Specifically, male ever smokers who had no education or were being illiterate smoked for 14% (95%*CI*: 1.00–1.31) longer compared with those who had college or above education. Male ever smokers who were service workers, office staff, manual and skilled workers, and farmers smoked for 26% (95%*CI:*1.06–1.50), 24% (95%*CI*: 1.00–1.55), 24% (95%*CI:*1.07–1.44) and 16% (95%*CI:*1.02–1.32) longer compared with those with professional or administrative jobs. Male ever smokers who earned the lowest income smoked for 11% (95%*CI*: 1.01–1.23) longer than those who earned the highest income. The magnitude of the association between education and smoking duration reduced largely in the adjusted log-logistic model. Further analysis (results not shown) indicated that the association between education and smoking duration was still significant after controlling for income and other covariates, but it was largely explained away when adding occupation in the model. Moreover, a male ever smoker’s age, age of smoking initiation and chronic conditions had a strong positive association with his length of smoking.

**Table 2:** Log-logistic Regression Models Estimating the Association between SES and Duration of Smoking among Male Ever Smokers Aged 18+, CHNS, 2006 (N = 2,637).

**Variables**	**Crude time ratios^a^ (95% *CI*)**	**Adjusted time ratios** **(95% *CI*)**
Age (years)		
18–24	1.00	1.00
25–34	2.37 (1.74–3.22)***	2.39(1.79–3.17)***
35–44	4.06 (3.01–5.47)***	4.30 (3.25–5.69)***
45–54	5.07 (3.79–6.78)***	5.72 (4.33–7.54)***
55–64	5.20 (3.90–6.93)***	6.07 (4.61–7.98)***
>=65	5.73 (4.30–7.64)***	6.88 (5.21–9.09)***
Marital status		
Married	1.00	1.00
Unmarried	1.04 (0.91–1.20)	1.02 (0.93–1.13)
Registration status		
Urban	1.00	1.00
Rural	1.24 (1.14–1.35)***	1.06 (0.98–1.14)
Region		
Coastal	1.00	1.00
Northeast	0.95 (0.84–1.08)	1.00 (0.92–1.09)
Inland	1.02 (0.92–1.14)	1.01 (0.93–1.09)
Mountainous south	1.15 (1.02–1.30)*	1.07 (0.98–1.16)
Education		
No education or illiterate	1.59 (1.37–1.85)***	1.14 (1.00–1.31)*
Primary	1.41 (1.21–1.64)***	1.07 (0.94–1.21)
Junior high	1.27 (1.10–1.47)**	1.02 (0.91–1.15)
Senior high	1.13 (0.97–1.33)	1.02 (0.90–1.14)
College or above	1.00	1.00
Occupation		
Farmer	1.56 (1.34–1.83)***	1.16 (1.02–1.32)*
Manual and skilled worker	1.35 (1.10–1.66)**	1.24 (1.07–1.44)**
Service worker	1.52 (1.19–1.95)***	1.26 (1.06–1.50)**
Office staff	1.26 (0.91–1.75)	1.24 (1.00–1.55)*
Professional and administrator	1.00	1.00
Other occupations	1.20 (0.94–1.54)	1.07 (0.91–1.27)
Unemployed	1.41 (1.21–1.65)***	1.08 (0.95–1.23)
Retired	1.27 (1.09–1.47)**	1.14 (1.01–1.28)*
Income quartiles		
Quartile 1	1.34 (1.19–1.51)***	1.11 (1.01–1.23)*
Quartile 2	1.23 (1.09–1.38)***	1.05 (0.96–1.15)
Quartile 3	1.04 (0.93–1.15)	0.96 (0.89–1.04)
Quartile 4	1.00	1.00
Age of smoking initiation (years)		
<=18	1.31 (1.16–1.47)***	1.39 (1.28–1.51)***
19–24	1.21 (1.10–1.34)***	1.23 (1.14–1.32)***
>=25	1.00	1.00
Chronic disease		
Yes	1.20 (1.08–1.33)***	1.10 (1.03–1.19)**
No	1.00	1.00
Self rated health		
Good/excellent	1.00	1.00
Fair/poor	0.99 (0.91–1.08)	0.95 (0.90–1.01)

^a^ Time ratios were computed by exponentiating the log-logistic regression coefficients.

*p<0.05, **p<0.01, ***p<0.001

## Discussion

This was one of the first studies to examine duration of smoking and the association between SES and length of smoking among Chinese population. The study demonstrated the problem of long smoking duration and a pattern of social disparities in smoking duration among Chinese male ever smokers.

The findings showed that male ever smokers aged 18 years and older had a median smoking duration of 58 years. Chinese male smokers experienced a much longer duration of smoking than smokers in Western countries. For example, male ever smokers in the United States and Australia have a median duration of smoking of 29 and 26 years, respectively. These lengths are about half of what Chinese male ever smokers have experienced [16, 18]. Consistent with other research on China [23, 29], our findings suggest that Chinese male ever smokers smoke until very old age. Furthermore, largely in support our hypotheses, the study showed that male smokers who were socially and economically disadvantaged had a longer duration of smoking after controlling for demographic characteristics, smoking history, and health status. While occupation had a strong negative association with duration of smoking, the inverse association between education, income and smoking duration was modest. The differential effect of various indicators of SES on smoking duration may suggest that different dimensions of SES may affect individual smoking behavior through different mechanisms.

Similar to Western literature, we found that male smokers who had a lower status job (i.e. service worker, office staff, manual and skilled worker, and farmer) had a significantly longer duration of smoking than those with a professional or administrative job, after controlling for the effect of education, income, and other covariates. We provide two possible explanations for the strong association between occupation and smoking duration among Chinese male ever smokers. Similar to what is found in other countries, work environment factors, such as work-related psychosocial stress and physical job strain, may also encourage smoking in China [30]. Besides, work environment may play an important role in allowing smoking culture to flourish in Chinese setting. In traditional Chinese culture, smoking was a means of social connection and leisure-time socialization. People used to consume tobacco together with friends or business partners in tobacco house or at home [31]. The norm of social smoking has been widely accepted and retained [19, 21, 32, 33, 34]. For example, cigarette gifting is highly visible among friends or colleagues, particularly among male smokers of lower SES groups [35, 36, 37]. There is an increasing tendency for individuals of lower occupational status to use cigarettes as a means for social networking [21].

The study also showed that male ever smokers who were in the lowest income group had a longer duration of smoking relative to those in the highest income group. There is strong evidence that the level of nicotine dependence is high among low income group. For example, studies in Western countries show that individuals with financial stress are more likely to smoke than their counterparts without financial stress [38, 39, 40]. This may also the case for the lowest income group in China. However, in transitional China, individuals of higher earnings, particularly male bread earners, may also experience higher level of nicotine dependence due to high level of psychosocial stresses. This may contribute to the modest association between income and length of smoking among male adult smokers in China.

The present study indicated a modest association between education and longer duration of smoking. This pattern is inconsistent with studies in Western countries, which show a strong educational gradient in smoking duration [16, 18]. The results may be partly due to the relatively low level of smoking-related health knowledge and risk perceptions among Chinese. A nationwide survey reveals that a large proportion of Chinese population know little of the specific hazards of tobacco use, which may possibly lead to their under-estimating of the health hazards of smoking [41]. Moreover, although better educated people are more knowledgeable about health risks of smoking, they may not be able to quit smoking due to the pervasive social pressure on smoking [42]. Our further analysis showed that the negative association between education and smoking duration was largely explained away by occupation. The finding may possibly suggest that the strong culture of social smoking among male smokers in lower social class overrides the influence of education.

The study has several limitations. First, the analysis is based on cross-sectional data, thus causal relationship between SES and duration of smoking cannot be determined. Second, the study may suffer from survivor bias. While participation is conditioned upon survival to enroll in the study, smokers of low SES are more likely to die prematurely, which may possibly lead to underestimation of the observed inverse association between SES and duration of smoking. Third, we relied on self-reported data to determine smoking-related outcome variables, which are likely to have misreporting and recalling bias. Previous studies show that self-reported data has not shown systematic differentials in under reporting of smoking status by socio-economic groups [43, 44, 45]. However, in an analysis of the extremely long average smoking duration, it is very likely to have recalling errors on age of smoking initiation and smoking cessation. Therefore, the results should be interpreted with caution. Finally, smoking is highly related to diseases such as lung cancer, chronic obstructive pulmonary diseases and lung infection. Unfortunately, CHNS did not collect this information. Without controlling for the important confounding effect of these co-morbidities, the association between SES and smoking duration may be overestimated.

Our study contributes significantly to the existing body of literature on the relationship between SES and smoking in china. The findings add another dimension by showing the problem of long smoking duration and a pattern of social disparities in length of smoking among Chinese male ever smokers. China has not only one of the highest smoking prevalence in the world, but also one of the longest durations of smoking. Longer duration of smoking is more apparent among male smokers of lower SES. If social disparities in smoking behavior continue to enlarge, it may exacerbate the already existing social inequalities in health.

The study has several policy implications. First of all, given the extremely long duration of smoking among Chinese male smokers, it’s very urgent for the government to enforce more aggressive tobacco control policies to support smoking cessation. Second, the public health campaigns to combat tobacco use should be implemented through multiple channels, and more importantly, to pay more attention to the disadvantaged groups. A multi-purpose intervention programs aimed at improving knowledge about the harmful effects of smoking as well as the skills in smoking cessation, stress management, and refusing cigarette gifting may be more effective in changing individuals’ smoking behaviors. Finally, as smoking behavior appears to be socially and culturally driven in China, future prevention or intervention programs should pay more attention to social determinants of smoking such as poor education, financial stress, and occupation-based pattern of social smoking.
